# Quality of life among Greek smokers and nonsmokers. A study in local community workers in Athens suburbia

**DOI:** 10.1186/1617-9625-12-S1-A33

**Published:** 2014-06-06

**Authors:** Ioannis Roxanis, Sotiria Makaroni, Maria Ginieri-Coccossis, Aggeliki Triantafyllou, Maria Typaldou

**Affiliations:** 1 First Department of Psychiatry, Eginition Hospital, Athens University Medical School, Athens, 11528, Greece; 2 Center for the Prevention of Addictions and Psychosocial Health Promotion "PRONOI", Athens, 14500, Greece; 3 Department of Biological Chemistry, Athens University School of Medicine, Athens, 10679, Greece

## Background

Smoking is a predictor of quality of life and smoking habits affect in different ways the quality of life between men and women. The aim of this study is the assessment of quality of life of a working population including smokers and non-smokers.

## Materials and methods

The WHOQOL-BREF has been used in a random sample of 144 municipal servants in a cross-sectional study.

## Results

46% of the study population were smokers. Independent samples t-test revealed no significant difference between how smokers and nonsmokers reported the main domains of their quality of life. However, smoking had significant impact on two specific parameters. Smokers recorded significant lower scores (3.62) than nonsmokers (3.86) in satisfaction from overall health, (p=0.04). Furthermore, smokers had statistically significant lower scores (3.30) in satisfaction from sleep than nonsmokers (3.68), (p=0.02). There was no significant difference between men and women smokers’ scores in main domains of quality of life, but for particular items concerned satisfaction from overall health (p=0.04) and difficulties from physical pain (p=0.00). Women smokers reported lower scores in both items than male smokers. Multiple regression analysis revealed that satisfaction from overall health had significant correlation (p=0.02) only with smoking and no other variables from those which have been examined.

## Conclusions

Smoking seems to affect quality of life as far as satisfaction from health is concerned. Further research, in bigger samples of working population may reveal correlations between smoking and more aspects of everyday life and more differences between male and female smokers.

